# Supplementary material for: Enhancement of the activity of the antimicrobial peptides HNP1 and LL-37 by bovine pancreatic ribonuclease A
Source: F1000Res. 2023 Mar 13;11:933. Originally published 2022 Aug 15. [Version 3] doi: 10.12688/f1000research.123044.3 (PMC10285323; doi:10.12688/f1000research.123044.3)
Supplement: Peer review history

# Peer review: Reviewer response for version 3

**DOI:** 10.5256/f1000research.145427.r174366

**Published:** 2023-06-06

**Authors:** Paweł M. Krzysciak

**Affiliations:** 1 Department of Infections Control and Mycology, Jagiellonian University Medical College, Kraków, Poland

The provided text discusses the challenges in developing cationic antimicrobial peptides (CAPs) as effective antibiotics and proposes a hypothesis regarding the inoculum effect observed when testing the defensin HNP1 against high bacterial cell concentrations. The text suggests that the decrease in efficacy at high cell concentrations could be attributed to the presence of defensin inhibitors, particularly polyanions like tRNA, which may bind and inhibit CAPs through electrostatic attraction.

 Overall, the text presents an interesting hypothesis that adds to our understanding of the factors influencing the efficacy of CAPs. The identification of potential inhibitors, such as polyanions, and their impact on antimicrobial peptide activity is a valuable contribution to the field. The proposed role of RNA inhibition, potentially not limited to tRNA, in the inoculum effect adds depth to the discussion.

 I suggest that  to address the all reviewers' concerns and strengthen the conclusions, it is recommended to conduct additional analyses using standard microbiological methods, such as colony-forming unit (CFU) counting or metabolic tests, such as the MTT assay. These analyses would provide complementary data to the Virtual Colony Count (VCC) assay and enhance the reliability and comprehensiveness of the findings.

 It would be beneficial to discuss the potential limitations associated with using TSB in the experimental setup and acknowledge the need for further investigations to validate the observed effects in a more controlled environment.

 The assumption made regarding the presence of biofilms based solely on the observation of cell clumps at 10x magnification should be addressed more cautiously. Biofilm formation is a complex process and typically requires specific conditions and timeframes. Without conducting specific biofilm assays or providing further evidence, it is uncertain whether the observed cell clumps can be definitively classified as biofilms. Acknowledging the limitations and potential alternative explanations for the observed clumps would strengthen the validity of the conclusions drawn from this observation.

 One area of improvement could be the inclusion of gene expression analysis related to biofilm formation. Since biofilm formation is a complex process involving the regulation of specific genes, it would be valuable to assess the expression levels of key biofilm-related genes. This could provide insights into the potential role of the observed cell clumps in biofilm formation and their relationship to the inoculum effect. Including gene expression analysis would strengthen the study by providing a molecular understanding of the observed phenomenon.

 It would be beneficial to provide more comprehensive statistical information, such as the t statistic value (or other suitable statistical test), degrees of freedom (df), power of the test, and the sample size (n) for each comparison made. These details are essential for the readers to evaluate the strength and reliability of the statistical analysis performed. By including the t statistic value and degrees of freedom, the readers can assess the magnitude of the observed differences and the precision of the estimates. Additionally, reporting the power of the test would provide insights into the sensitivity of the statistical analysis to detect true differences if they exist. Lastly, providing the sample size (n) would give readers an understanding of the reliability of the observed results.

 In summary, by incorporating the suggested improvements, including additional microbiological analyses, addressing limitations, considering alternative explanations, and providing comprehensive statistical information, the text would make a valuable contribution to the field of antimicrobial peptide research. The proposed hypothesis and insights into the inoculum effect and potential inhibitory factors enhance our understanding of CAPs' efficacy as antibiotics and pave the way for future investigations in this area.

Is the work clearly and accurately presented and does it cite the current literature?

Yes

If applicable, is the statistical analysis and its interpretation appropriate?

Partly

Are all the source data underlying the results available to ensure full reproducibility?

Yes

Is the study design appropriate and is the work technically sound?

Yes

Are the conclusions drawn adequately supported by the results?

Yes

Are sufficient details of methods and analysis provided to allow replication by others?

Yes

Reviewer Expertise:

medical microbiology, medical mycology, antimicrobial susceptibility testing,

I confirm that I have read this submission and believe that I have an appropriate level of expertise to confirm that it is of an acceptable scientific standard, however I have significant reservations, as outlined above.

# Peer review: Reviewer response for version 3

**DOI:** 10.5256/f1000research.145427.r166245

**Published:** 2023-03-27

**Authors:** Ramakrishnan Nagaraj

**Affiliations:** 1 Centre for Cellular and Molecular Biology, Hyderabad, India

The authors have adequately answered my recommendations

Is the work clearly and accurately presented and does it cite the current literature?

Partly

If applicable, is the statistical analysis and its interpretation appropriate?

Not applicable

Are all the source data underlying the results available to ensure full reproducibility?

Yes

Is the study design appropriate and is the work technically sound?

Partly

Are the conclusions drawn adequately supported by the results?

No

Are sufficient details of methods and analysis provided to allow replication by others?

Partly

Reviewer Expertise:

NA

I confirm that I have read this submission and believe that I have an appropriate level of expertise to confirm that it is of an acceptable scientific standard.

# Peer review: Reviewer response for version 2

**DOI:** 10.5256/f1000research.141606.r163610

**Published:** 2023-03-07

**Authors:** Ramakrishnan Nagaraj

**Affiliations:** 1 Centre for Cellular and Molecular Biology, Hyderabad, India

The brief report titled “Enhancement of the activity of the antimicrobial peptides HNP1 and LL-37 by bovine pancreatic ribonuclease A” by Ericksen describes the effect of tRNA and RNase A on antibacterial activity of HNP1, HBD1 and LL37 at low and high inoculum by VCC assay. Activity of HNP1 is inhibited by tRNA at 1:1 but restored when RNase A is present at 25:1. Similar effects are not observed with LL37. RNase A does not modulate the activity of HBD1 at high inoculum. The fact that inhibition of activity was observed at high inoculums, but not when RNase is present, suggesting that peptide binds to tRNA, is interesting. It is interesting as it could provide insights into HNP1 inactivation. However, more definitive experiments to confirm this result are necessary and worth doing.

 Points

1. The author argues that RNase 25:1 to HNP1 enhanced activity at high inoculum. It appears that the antibacterial activity of HNP1 that is abrogated at high inoculum is restored not enhanced. Antibacterial activity is restored in the presence of tRNA and RNase A.
2. Wherever the activity is restored, antibacterial activity is restored abruptly (Figs 2, 5,6) unlike in controls (Figs 1, 3, 4).
3. The presence of tRNA in the medium under high inoculum has not been demonstrated.
4. The concentrations should have been represented as molar concentrations.
5. DNA could also inhibit antibacterial activity which has not been demonstrated.
6. The enzyme activity of RNase A could have been determined in 10 mM sodium phosphate buffer. There is too much speculation in the Conclusion section. For example, “It is unknown whether the RNA-degrading activity of RNase or its cationicity is responsible for the enhancement of HNP1 and LL-37 activity”.
7. As the author states “Further studies are warranted to determine whether these results are biologically relevant or could be generalized to antimicrobial peptide-nuclease combinations, as might be suggested by the presence of DNA in biofilms”.

The data are preliminary and more definitive experiments are necessary. There is no enhancement in activity in the presence of RNase A, only restoration of activity. The author should demonstrate the presence of bacterial tRNA in high inoculum and also should address more conclusively the varied effects on LL37 and HBD1. The author should provide details of synthetic HNP1 characterization to rule out any trivial explanation.

Is the work clearly and accurately presented and does it cite the current literature?

Partly

If applicable, is the statistical analysis and its interpretation appropriate?

Not applicable

Are all the source data underlying the results available to ensure full reproducibility?

Yes

Is the study design appropriate and is the work technically sound?

Partly

Are the conclusions drawn adequately supported by the results?

No

Are sufficient details of methods and analysis provided to allow replication by others?

Partly

Reviewer Expertise:

Biology of host-defence peptides such as defensins, peptide antibiotics, mechanisms of bacterial killing by defensins.

I confirm that I have read this submission and believe that I have an appropriate level of expertise to confirm that it is of an acceptable scientific standard, however I have significant reservations, as outlined above.

# Response

**Published:** 2023-03-09

**Authors:** Bryan Ericksen

**Affiliations:** University of Maryland, USA

Dear Dr. Nagaraj,

 Thank you for your detailed review.  I will respond to each of your points.

1.  The more general term “enhanced” is more appropriate than “restored”, because the activity of HNP1 at the standard inoculum is far greater than the activity of HNP1 + 25x RNase at the high inoculum. Therefore, we cannot say that activity has been fully “restored” but it has certainly been “enhanced” by RNase.
2. I have added the word “abruptly” in the Results section, description of Figure 2.
3. The sentence “However, the presence of tRNA in the medium at the high inoculum has not been demonstrated” has been added to the Discussion section.
4. There is a long history of expressing concentrations as µg/mL instead of µM. Please refer to the seven Journal of Biological Chemistry publications referenced in the Ericksen 2020 reference.
5. DNA could certainly inhibit antibacterial activity, as I speculated in the Discussion section. I hope that speculation will lead another researcher to test DNA+RNA+antimicrobial peptide combinations.
6. I apologize for neglecting to demonstrate the enzyme activity of RNase in 10 mM sodium phosphate buffer pH 7.4, but I feel it Is reasonable to speculate that the enzyme would be active under these conditions, especially given its structural robustness.
7. Once again, I hope my speculation encourages another researcher to test DNA in experiments like these.

# Peer review: Reviewer response for version 2

**DOI:** 10.5256/f1000research.141606.r157025

**Published:** 2023-01-25

**Authors:** Bouke K. H. L. Boekema

**Affiliations:** 1 Department of Plastic, Reconstructive and Hand Surgery, Amsterdam Movement Sciences Amsterdam UMC, Amsterdam, The Netherlands; 2 Preclinical Research, Association of Dutch Burn Centres (ADBC), Beverwijk, The Netherlands

Concerning Figure 2, 5 and 6: you state that standard deviations have been added but this is not the case. If I understand you correctly, the results shown in these figures are single values. These must be averaged in 1 figure. There is no added value of showing them separately. The excel file is missing, but there should be  raw data (or a back-up of the raw data), which can be used to generate a new figure?

 "Evidence that tRNA induces biofilm is the observation of cell clumps", then you should mention clumping and not biofilm.

Is the work clearly and accurately presented and does it cite the current literature?

Partly

If applicable, is the statistical analysis and its interpretation appropriate?

No

Are all the source data underlying the results available to ensure full reproducibility?

Partly

Is the study design appropriate and is the work technically sound?

Partly

Are the conclusions drawn adequately supported by the results?

Yes

Are sufficient details of methods and analysis provided to allow replication by others?

Partly

Reviewer Expertise:

Microbiology, wound infection, antimicrobial peptides/therapies.

I confirm that I have read this submission and believe that I have an appropriate level of expertise to confirm that it is of an acceptable scientific standard, however I have significant reservations, as outlined above.

# Response

**Published:** 2023-03-09

**Authors:** Bryan Ericksen

**Affiliations:** University of Maryland, USA

Dear Dr. Boekema,

 Thank you for suggesting adding a figure showing the mean of Figures 2, 5 and 6.  Figure 9 also shows the standard error of the mean. I do believe that there is added value showing the figures separately, as it allows the reader to grasp fully the variation between experiments.

 Regarding mentioning clumping and not biofilm, the word biofilm has been removed from the abstract. In addition, the sentence, “It should be emphasized that these experiments do not prove that the observed cell clumps are biofilms” has been added to the discussion. I should mention that subsequent experiments in the laboratory of Dr. Wuyuan Lu strongly suggest that cell clumps observed in VCC assays are in fact biofilms. Those experiments have yet to be published.

# Peer review: Reviewer response for version 1

**DOI:** 10.5256/f1000research.135107.r155649

**Published:** 2022-11-18

**Authors:** Bouke K. H. L. Boekema

**Affiliations:** 1 Department of Plastic, Reconstructive and Hand Surgery, Amsterdam Movement Sciences Amsterdam UMC, Amsterdam, The Netherlands; 2 Preclinical Research, Association of Dutch Burn Centres (ADBC), Beverwijk, The Netherlands

The idea of enhancing antimicrobial activity (of AMPs) is appealing and might be extended to other molecules/antimicrobials. Although there is a clear effect of tRNA on bacterial survival, the current paper is lacking in depth and does not meet the standards of scientific reporting. The introduction, materials & methods and results sections are rather limited. By what mechanisms might tRNA and RNase influence the AMP activities? There are several options which need to be addressed and experimentally tested.

 The low number of AMPs approved for clinical use is mainly due to i) unfavorable pharmacokinetic profile, ii) safety issues and iii) reduced activity in clinically relevant environments. In view of this, the limited activity of the AMP-RNase combination does not show high potential.

**Minor Issues**

 When treating more bacteria, it seems only logical that more antibacterial is required. Is it  biologically relevant if there is only bactericidal activity in case of low level of bacteria?

 An alternative for VCC would be the use of a microcalorimeter for real time measurements.

 The bacterial species is not mentioned in MM.

 Why was the percentage TSB varied? And how would TSB affect activity?

 The ratio tRNA to HNP, is this in mol/mol?

 Addition of RNase 25:1 to HNP1 enhanced activity (figure 2) but only at the highest concentration of HNP1.

 ‘LL-37 was enhanced’ (Figure 4)? Meant is that LL37 activity was enhanced? But in fig 4 more LL37 is needed for same effect as in fig3 (so it is not enhanced)? Please check/explain.

 ‘RNase was not active in the absence AMP’: this is probably not true. RNase will still be active (against RNA) but not detectable as antimicrobial.

 For all Figures: how many replicates were used, where is standard deviation/statistics?

 Figure 3: where are the results with HBD1 without tRNA?

 There is mention of using washed cells but it is not clear how this was done (timing of washing).

 The conditions for biofilm formation seem rather random, please elaborate.

 ‘possible that tRNA inducing biofilm’: is there evidence for this?

 Furthermore, I fully agree with the comments by reviewer Jacobs.

 In case bacteria secrete peptide inactivating substances this can be tested by using culture supernatants.

 Restructuring the graphs would improve presentation of the results.

 There is a clear effect of tRNA on bacterial killing, which might be demonstrated more clearly by using for example LIVE/DEAD stain, which does not involve dilution and culture and can be used on both low and high bacterial concentrations.

Is the work clearly and accurately presented and does it cite the current literature?

Partly

If applicable, is the statistical analysis and its interpretation appropriate?

No

Are all the source data underlying the results available to ensure full reproducibility?

Partly

Is the study design appropriate and is the work technically sound?

Partly

Are the conclusions drawn adequately supported by the results?

Yes

Are sufficient details of methods and analysis provided to allow replication by others?

Partly

Reviewer Expertise:

Microbiology, wound infection, antimicrobial peptides/therapies.

I confirm that I have read this submission and believe that I have an appropriate level of expertise to state that I do not consider it to be of an acceptable scientific standard, for reasons outlined above.

# Response

**Published:** 2022-11-26

**Authors:** Bryan Ericksen

**Affiliations:** University of Maryland, USA

Dear Dr. Boekema,

 Thank you for your detailed comments. I will respond to each paragraph point by point in the same order as in your reviewer report.

 First paragraph:

 Although this article is lacking in depth and does not include mechanistic experiments, it is merely a Brief Report, intended as an initial study of antimicrobial activity only. Although the Introduction, Methods, and Results sections are limited, version 1 was very close to the 2500 word limit for a Brief Report. I cannot expand these sections without going over the word limit. I feel a Brief Report is appropriate because the results of only one method, VCC, are presented. 

 Second paragraph:

 Although the activity of antimicrobial peptides in combination with RNase is slight, I feel it is significant because any activity at the high inoculum could be the difference between a lethal infection and an infection that the immune system has time to control in vivo, especially given the lack of activity of HNP1 alone at the high inoculum. Furthermore, these results are a stepping stone to antimicrobial peptide+DNase+RNase combinations, which may have statistically significant activity. As an initial study of an interesting phenomenon these results deserve to be published.

 Minor issues:

 Further studies are required to determine whether the amount of activity reported here is biologically relevant. I added the words, “are biologically relevant or” to the first sentence in the last paragraph of the Conclusions section.

 I agree microcalorimetry can be used to detect antibacterial activity. I would look forward to a report by another researcher using such a method to confirm these results. 

 “Of E. coli ATCC 25922” was added to the Methods section.

 The percentage of TSB was varied because it has two opposite effects. The nutrients increase activity, whereas the added salt decreases activity. I had been using 1% TSB for many years as instructed by Robert I. Lehrer, the original developer of the VCC assay, but I never tested other TSB concentrations until this article. I feel the results of the TSB variation are of interest to the study of defensins and so I included them in this article.

 The ratio of tRNA to HNP1 is wt/wt, not molar. I added “wt/wt” to the Abstract.

 I added “at the highest tested concentration of HNP1” to the Abstract.

 The difference between Figure 3 and Figure 4 is that Figure 3 is at the standard inoculum, whereas Figure 4 is at the high inoculum. Therefore, Figure 4 does show an enhancement of activity due to the addition of RNase at the high inoculum. I added the word “activity” before enhanced in the Results section.

 I replaced the word “active” with “antimicrobial” regarding RNase alone.

 Figures 2, 5 and 6 can be regarded as triplicate experiments, as I mentioned in the response to Reviewer 1. Standard deviations and p-values have been added.

 The results of HBD1 without RNA were not tested because there was insufficient room on the 96-well plate. I apologize for the mistreatment of HBD1 in this article. In general, the emphasis of the article is on HNP1 more than LL-37, and LL-37 more than HBD1. Further studies of HBD1 are warranted. I considered omitting the HBD1 results but I included them for completeness.

 Because data are not shown, the mention of washed cells has been deleted.

 Biofilms formed at high inocula under the standard conditions of VCC assays. See the Ericksen 2020 reference for a further discussion. I added “similar to those previously described (Ericksen 2020)” to the Results section.

 The evidence that tRNA induces biofilm is the observation of cell clumps in the 96 well plate after the assay. I believe this evidence is clear from the article as is.

 I have fully responded to the comments of Reviewer 1.

 I agree testing culture supernatants would be interesting. I look forward to a future article presenting such results.

 There is a long history of this style of graph in VCC articles. For example, see the seven Journal of Biological Chemistry articles cited in the Ericksen 2020 reference. In any case, the hard drive of the computer containing the Excel files failed, so these figures cannot be remade.

 I agree that further experiments using LIVE/DEAD stain in conjunction with flow cytometry would be interesting, although well beyond the scope of this article, which is intended as an initial study using just the VCC method.

# Peer review: Reviewer response for version 1

**DOI:** 10.5256/f1000research.135107.r147551

**Published:** 2022-09-07

**Authors:** William R. Jacobs

**Affiliations:** 1 Department of Microbiology and Immunology, Albert Einstein College of Medicine, New York, NY, USA

Hi Dr. Erickson,

             Thank you for sharing this research with us. The idea of using antimicrobial peptide-ribonuclease combinations could be beneficial for use when the microbial agent alone is ineffective, such as when used against high bacterial concentrations and biofilms. However, the virtual colony count has many caveats that would make the data not reflective of the true killing effect of the peptides. For this paper, why not simply use colony forming units? The author hypothesizes that higher densities of bacteria cause inactivation of the peptides by secretion of tRNAs, but provides no data substantiating this hypothesis. The author could have taken the supernatant fluid from the more densely populated culture and identified compounds that inactivated the peptides. An alternative hypothesis is that the bacterial cells are entering into a persistent state where they are expressing a genetic program that prevents killing by the peptides. Overall, the data is convincing that tRNA inhibits peptide activity, but it’s unclear that this has real world relevance. The author does show a modest effect of peptide-ribonuclease combinations on high inoculum cultures, but the VCC is an inadequate measure of killing. I believe that parts of this manuscript should be reworked and more data should be obtained to prove the effectiveness of these combinations.

 Minor Comments:

1. In the results section of the abstract I think the ordering could be more streamlined. Currently it goes through all the results for HNP1 and then goes through the HBD1 and LL-37 results together and I am not sure this is the most effective way to convey this information succinctly, especially seeing it is mostly pulled as written from the beginning of the results section. An alternative way would be to describe all three together.
2. In methods “HNP1, LL-37 and HBD1 were synthesized ABI 433A synthesizer using an optimized” may read better as “were synthesized with an ABI 433A synthesizer.”
3. In some figures, especially 2, 5, and 6, the different shapes are difficult to parse. There’s a bunch of small, similar looking, overlapping shapes, and the way the shapes look on the key do not always perfectly correspond to the graph. For example, in Figure 2 in the key 25x RNase looks like a single uptick but I’m pretty sure from looking at the graph section it’s actually a plus sign. I think that if you write the inoculum (with CFUs/mL not just high or standard) in the figure title you don't need it in the key. It just makes things more cluttered to look at and harder to read for the relevant information differentiating each line. If you disagree that’s fine, but it should be consistent: Figure 1 doesn’t have CFUs/mL in the key, Figure 2 does, Figure 3 does for some of the conditions, etc.
4. Figure 3 is called “LL-37 was assayed at the standard inoculum with or without tRNA,” but this is also where you show your HBD1 tRNA data so the title should reflect that. Also you should show how effective HBD1 is before tRNA is used for inhibition especially since you say that tRNA is abrogating the HBD1 activity but I’m not seeing what the activity looked like originally. I have a similar issue with Figure 4 where I think the HBD1 data without RNase should be given to match LL-37.
5. You write that a “pronounced inoculum effect was also observed when HNP1 was assayed against Staphylococcus aureus ATCC 29213 and Bacillus cereus ATCC 10876.” Do you believe that doing the RNase and tRNA experiments with these bacteria would show similar results?
6. You write that “the enhancement of activity caused by RNase was observed with LL-37 but not HNP1 when washed cells were used, indicating that RNase operates by different mechanisms with the two antimicrobial peptides” I think you must mean HBD1 instead of HNP1 or what I am getting from your graphs is wildly different from what you are. Additionally, I don’t see a difference between LL-37 alone vs LL-37 with 25x RNase in Figure 4 in anything besides the highest peptide condition, and there’s nothing on any of your graphs that mark differences as statistically significant.
7. What mechanism would you think the RNase improves LL-37 activity by since it is much less impacted by tRNA and high inoculum as the defensins so there’s less of a problem for the RNase to solve. Additionally, it would be good to show RNase either reversing or failing to reverse the effect of tRNA on HBD1 like you do for HNP1, especially if you want to claim that RNase is not enhancing HBD1 activity.
8. Regarding mechanism, has the author isolated mutants of the bacterial strains that are resistant to peptide function? A rigorous genetic analysis could provide important insights into the peptides specificities and functions.
9. Further, you use a log scale for experiments done at a standard inoculum, but not for those at a high inoculum, so it seems clear that even when using the RNase, these peptides are orders of magnitude less effective at high bacterial concentrations. Is a 30% decrease in virtual survival at the highest peptide concentration enough to consider these combinations effective when compared with non-CAP antibiotics?

Is the work clearly and accurately presented and does it cite the current literature?

Partly

If applicable, is the statistical analysis and its interpretation appropriate?

No

Are all the source data underlying the results available to ensure full reproducibility?

No

Is the study design appropriate and is the work technically sound?

No

Are the conclusions drawn adequately supported by the results?

No

Are sufficient details of methods and analysis provided to allow replication by others?

No

Reviewer Expertise:

Microbial genetics, tuberculosis, leprosy, herpes.

I confirm that I have read this submission and believe that I have an appropriate level of expertise to state that I do not consider it to be of an acceptable scientific standard, for reasons outlined above.

# Response

**Published:** 2022-09-08

**Authors:** Bryan Ericksen

**Affiliations:** University of Maryland, USA

Dear Dr. Jacobs,

 You have not provided any rational reason for your prejudice against the virtual colony count (VCC) assay. You suggest using the colony count assay, but VCC has one major advantage: in the colony count assay, cells must be diluted after exposure to antimicrobial agents in order to result in 30-300 colonies per plate. This dilution is problematic when cells are assayed at the standard inoculum, but much more so at a high inoculum. If an inoculum of 6.25x10^7 CFU/mL is used, and survival is .5, then cells must be diluted 5 orders of magnitude to reach the range of 30-300 colonies per plate. This dilution series adds inaccuracy to the assay. In the VCC procedure, by contrast, cells are not pipetted after the exposure to antimicrobial agents. Instead, twice-concentrated Mueller-Hinton broth is added and the 96-well plate is read for 12 hours in a plate reader. The VCC assay is much more precise. Its published results since 2005 in journals such as Antimicrobial Agents and Chemotherapy and The Journal of Biological Chemistry have demonstrated that the assay is highly reproducible except for paradoxical points, as is once again demonstrated in the present work for the triplicate results of HNP1+RNase at the high inoculum, assuming both preparations of RNase are equivalent. I encourage you to read the Ericksen 2020 reference, which includes a review of VCC studies up to that point and provides a peer-reviewed precedent for the use of VCC at high inocula.

 Sincerely,

 Bryan Ericksen, Ph.D.

# Response

**Published:** 2022-09-30

**Authors:** William Jacobs

**Affiliations:** Albert Einstein College of Medicine, USA

Dear Bryan,

 Thank you for your thoughtful response. Our lab has done death curves on Mtb for the last 10 years to look at the presence of persisters when subjected to bacteriocidal antibiotics. Typically we see a biphasic death curve that either leads to sterilization or drug resistance. I am not understanding with your technique how you are distinguishing amongst the four population: 1. live cells from, 2. dead cells, 3. persisters, and 4. drug resistant cells. While I await your answer, I will review the paper you cited.

 Furthermore, I did a search on Pubmed for the paper and cannot find it, can you provide the PMID number.

 Best,

 Bill

# Response

**Published:** 2022-11-26

**Authors:** Bryan Ericksen

**Affiliations:** University of Maryland, USA

Dear Dr. Jacobs,

 Thank you for your detailed comments.  I will respond to each paragraph point by point.

 Initial paragraph:

 I am not sure what caveats you are referring to, but it is true that the VCC assay is sensitive not only to bactericidal killing but also to lag times.  Therefore, it can be regarded as an assay for general antibacterial activity, not killing, as is reflected in the title of the first VCC publication in 2005, “Antibacterial activity and specificity of the six human alpha defensins”.  It should be emphasized that the ability to cause bacterial cells to lag before growing could allow the rest of the innate and adaptive immune systems time to respond to the infection in vivo; therefore, both types of activity are significant.  VCC is an appropriate method for an initial study determining antibacterial activity, which can later be followed up by a study using the colony count assay determining bactericidal killing if a distinction is important between killing and causing lag times.  The advantages of VCC, such as using less media and the absence of a dilution series after exposure to the antimicrobial agent, make it ideal for a first study.  I feel the Ericksen 2020 reference adequately discusses all of these points and they do not need to be repeated in this publication.  As an initial study using only one method, I feel it is appropriate that this manuscript is a Brief Report, not a full-length article where one might see the results of multiple assay methodologies.  I would welcome a future publication by other researchers reporting colony forming units.  

 You mischaracterized my hypothesis somewhat.  I had to choose a convenient source of RNA for the study, so I chose tRNA, but my hypothesis is that any RNA, especially RNA normally secreted as part of bacterial growth or incorporated into biofilms, could inhibit antimicrobial peptides.  I do not mean to imply that tRNA is actively secreted by bacterial cells in a manner similar to other RNA, although undoubtedly some tRNA is released upon exposure to antimicrobial peptides due to the lysis of cells.  To emphasize this distinction, I have added “quite possibly a general property of RNA, not specific to tRNA” to the sentence where the hypothesis is first described in the introduction. In addition, in the conclusions section I have added, “Any RNA, especially RNAs normally secreted as part of bacterial growth or incorporated into biofilms, could inhibit antimicrobial peptides.”  Your alternative hypothesis is certainly also occurring simultaneously: persister cells that do not grow are not killed by defensins, as was demonstrated in the 1980s by the original developer of the VCC assay, Robert I. Lehrer.  I consider a study of the phenomenon of persistence to be beyond the scope if this Brief Report.  Please note that I wanted to acknowledge Dr. Lehrer for sharing with me an unpublished manuscript hypothesizing that RNA inhibits defensins, but he has retired and did not respond to my request for permission to name him in the Acknowledgments section.

 Minor Comments:

 1.    I am not sure what you mean by “describe all three together”.  Do you mean include the results from all three in one long sentence?  I believe the abstract is ideal as is, because results are presented in the same order as in the figures.  I especially think it is appropriate to go through all the results for HNP1 first, since HNP1 was the primary focus of this study, with more emphasis and more experiments than LL-37 or HBD1.

 2.    The words, “with an” were added to the methods section.

 3.    You are correct that one of the Microsoft Excel shapes is a plus sign.  The legend shows both the shape and the line; therefore, the horizontal part of the plus sign cannot be distinguished from the line.  I was consistent in that I used “standard” and “high” to describe the inocula in the figure titles but used the CFU/mL in the legend keys.  Having both types of description in the same figure gives all relevant information succinctly.  I apologize for leaving the CFU/mL off of the legends for Figure 1 and Figure 6.  Unfortunately, the hard drive of the computer containing the Excel files failed, so these figures cannot be remade short of reading each value off the plots and manually reentering the data.  There is a long history of using this style of figure to present VCC results, as you can appreciate if you look at the seven Journal of Biological Chemistry publications cited in the Ericksen 2020 reference, for example.

 4.    The title of Figure 3 has been edited to include “and HBD1 was assayed with tRNA.”  I apologize for the mistreatment of HBD1 in this study.  HBD1 was not assayed without tRNA in the experiment shown in Figure 3, nor was it assayed without RNase in the experiment shown in Figure 4, due to limited space available on the 96-well plate.  As such, the HBD1 data can be regarded as preliminary.  Further experiments using HBD1 would be of interest.  I considered omitting HBD1 from this manuscript but I decided to include it for completeness.  To improve the information presented on HBD1, I included “and HBD1+tRNA” in the Abstract sentence mentioning cell clumps and in the Results section regarding cell clumps.

 5.    Yes I expect this phenomenon is not limited to E. coli.  I look forward to future publications by other researchers testing other strains.

 6.    Because data were not shown, the mention of washed cells has been deleted.  Although statistics cannot be reported for the single experiments using LL-37, a standard deviation can be calculated for the triplicate experiments using HNP1 shown in Figures 2, 5, and 6, assuming the two RNase preparations are equivalent.   The following was appended to the Results section: “The results shown in Figures 2, 5, and 6 can be regarded as triplicate experiments, assuming the two RNase preparations are equivalent.  The mean virtual survival of HNP1 + 5x RNase was 0.75, and the standard deviation was 0.21.  The mean virtual survival of HNP1 + 25x RNase was 0.70, and the standard deviation was 0.10.  The mean virtual survival of HNP1 alone was 0.88, and the standard deviation was 0.02.  Based on these values, the two-tailed p-value for HNP1 + 5x RNase compared to HNP1 alone was 0.38, whereas the two-tailed p-value for HNP1 + 25x RNase compared to HNP1 alone was 0.10.  Therefore, the slight differences in activity observed were not statistically significant (p>0.05).”  Finally, the following was added to the Conclusions section: “Although the effect of HNP1+RNase was not statistically significant compared to HNP1 alone, it is possible that the effect of an antimicrobial peptide+DNase+RNase would be significant compared to the antimicrobial peptide alone.”

 7.    I prefer not to speculate too much regarding the mechanism in the main text of the publication, but there are probably two mechanisms at work here: direct binding of the anionic RNA to the cationic antimicrobial peptide, and indirect induction of biofilm by RNA, in which case it is the biofilm that inhibits the antimicrobial peptide, not RNA itself.  It is possible that both mechanisms are important for HNP1, but only the indirect mechanism is important for LL-37.  This explanation highlights the assumption that hydrophobic interactions must be important for RNA binding to antimicrobial peptides, since electrostatic charge cannot explain this difference.

 8.    I agree that a rigorous genetic analysis would be of interest and I hope the publication of this initial Brief Report encourages another researcher to undertake and publish those studies.

 9.    I agree that the observed differences are slight.  I included the word “slight” in the changes made in response to comment number 6, above.  A comparison with non-CAP antibiotics is beyond the scope of this study, but I would speculate that a 30% difference might be important simply because achieving any activity against biofilms at the high inoculum could be the difference between a lethal infection and an infection delayed such that the host immune system can mount an effective defense.  Any activity against biofilms might be important, especially given the minimal activity of HNP1 at the high inoculum.

# Response

**Published:** 2022-11-26

**Authors:** Bryan Ericksen

**Affiliations:** University of Maryland, USA

Dear Dr. Jacobs,

 The Ericksen 2020 reference is not indexed in Pubmed.  It can be found in the peer-reviewed journal WikiJournal of Science: WikiJournal of Science/Virtual colony count - Wikiversity

 Dr. Lehrer and I speculated in the initial VCC publication ( Antibacterial Activity and Specificity of the Six Human α-Defensins | Antimicrobial Agents and Chemotherapy (asm.org) that biphasic survival curves were due to the presence of persister cells.  They may also explain paradoxical data, some of which is presented in this publication.  However, optical density measurements in a plate reader cannot distinguish between actively growing cells and persisters.  The study of persisters is well beyond the scope of this Brief Report, which is intended as an initial study using VCC alone.
